# Real-time *ex-vivo* measurement of brain metabolism using hyperpolarized [1-^13^C]pyruvate

**DOI:** 10.1038/s41598-018-27747-w

**Published:** 2018-06-22

**Authors:** Talia Harris, Assad Azar, Gal Sapir, Ayelet Gamliel, Atara Nardi-Schreiber, Jacob Sosna, J. Moshe Gomori, Rachel Katz-Brull

**Affiliations:** Department of Radiology, Hadassah-Hebrew University Medical Center, Jerusalem, 9112001 Israel

## Abstract

The ability to directly monitor *in vivo* brain metabolism in real time in a matter of seconds using the dissolution dynamic nuclear polarization technology holds promise to aid the understanding of brain physiology in health and disease. However, translating the hyperpolarized signal observed in the brain to cerebral metabolic rates is not straightforward, as the observed *in vivo* signals reflect also the influx of metabolites produced in the body, the cerebral blood volume, and the rate of transport across the blood brain barrier. We introduce a method to study rapid metabolism of hyperpolarized substrates in the viable rat brain slices preparation, an established *ex vivo* model of the brain. By retrospective evaluation of tissue motion and settling from analysis of the signal of the hyperpolarized [1-^13^C]pyruvate precursor, the T_1_s of the metabolites and their rates of production can be determined. The enzymatic rates determined here are in the range of those determined previously with classical biochemical assays and are in agreement with hyperpolarized metabolite relative signal intensities observed in the rodent brain *in vivo*.

## Introduction

Magnetic resonance spectroscopy (MRS) of ^13^C-labeled substrates is an attractive approach to study brain metabolism as it can non-invasively quantify the flux of isotopic label in living tissues. It has been used to characterize cerebral metabolic fluxes in the normal brain^1–5^ as well as in disease models including diabetes^6,7^, traumatic brain injury^8^, and epilepsy^9^. However, ^13^C cerebral MRS spectroscopy *in vivo* is largely limited by the long measurement times required and the concomitantly large quantities of ^13^C-labeled materials necessary for such studies. The development of the dissolution dynamic nuclear polarization (dDNP) methodology by Ardenkjaer-Larsen *et al*.^10^ that can enhance the liquid state ^13^C NMR signal by four orders of magnitude, reduces the amount of labeled material and measurement times allowing a re-examination of cerebral MRS. Since the development of the dDNP technology, many studies have been published characterizing the cerebral metabolism of hyperpolarized ^13^C-labeled substrates *in vivo*^11–20^.

However, the metabolic phenotype observed in these studies may be convoluted by significant influx of metabolic products from other organs to the brain during the hyperpolarization acquisition window and by transport of metabolites across the blood brain barrier (BBB), necessitating the development of complex metabolic models to translate the observed hyperpolarized signal to metabolic rates^15,16,18^. Additionally, it has been shown that these *in vivo* studies are strongly affected by the anesthesia necessary for preclinical imaging, further complicating the characterization of metabolic rates^13^. For these reasons we were interested in developing a system to investigate the intrinsic metabolism of hyperpolarized substrates by the brain without the convoluting effects present *in vivo*. *Ex vivo* brain slices are an ideal model system for studying the metabolism of brain tissue, as these slices preserve the *in vivo* architecture of the brain but the metabolic rates observed will not be affected by the influx of metabolites from the periphery or the BBB transport^21–28^. In addition, in this preparation, the microenvironment of the brain tissue can be controlled and finely tuned, thus promising a new window to study the factors influencing brain metabolism and the potential effects of exogenous compounds.

To this end, we modified previously described systems for preparing rat brain slices and maintaining them in the NMR magnet^29,30^ to allow rapid injections of hyperpolarized metabolite solutions to the perfused brain slices. The system is designed to allow repeated injections of hyperpolarized metabolites, while the viability and energetics of the tissues are monitored between injections with ^31^P spectroscopy.

Studying the metabolism of hyperpolarized substrates in tissue slices can be complicated as the rapid injection, necessary for observing the metabolic signals within the hyperpolarized substrate lifetime, can result in displacement and then slow settling of the tissue slices in the active region of the NMR coil. In previously used bioreactor systems in which the metabolism of tumor cells grown on beads were studied^26,28^, this problem was avoided by fixing the beads in place by an immobile filter. However, here, the delicate brain slices could not withstand the mechanical pressure experienced when such a filter was used. In a couple of previous reports of hyperpolarized metabolic studies in perfused tumor tissue slices^27,31^, the authors do not report fitting the dynamic spectra to a kinetic model, but instead sum all spectra and consider the ratio of the total product to precursor signal and it is not reported whether this problem was experienced in that preparation as well. We describe here a general method to evaluate the extent and duration of tissue settling due to the rapid injection of hyperpolarized solution, when this occurs, and use this information to better characterize the metabolic rates. Using the experimental system and analytical approach described herein, we have characterized the cerebral metabolic rates of [1-^13^C]pyruvate in real time.

Pyruvate is found at the junction of anaerobic glycolysis and oxidative phosphorylation and understanding the interconnected metabolism of pyruvate, lactate, and bicarbonate in the brain has gained interest in the field of traumatic brain injury (TBI) and stroke^32^. We demonstrate that cerebral pyruvate metabolism rates can be determined within only 1–2 min of continuous monitoring, even in cases of sample motion, and show significant differences in the T_1_s of the different metabolites observed in this manner. Additionally, we show a correlation between the rates of pyruvate conversion to lactate and its conversion to bicarbonate.

## Results

### Validation of viability of the brain slices

**Figure 1:**
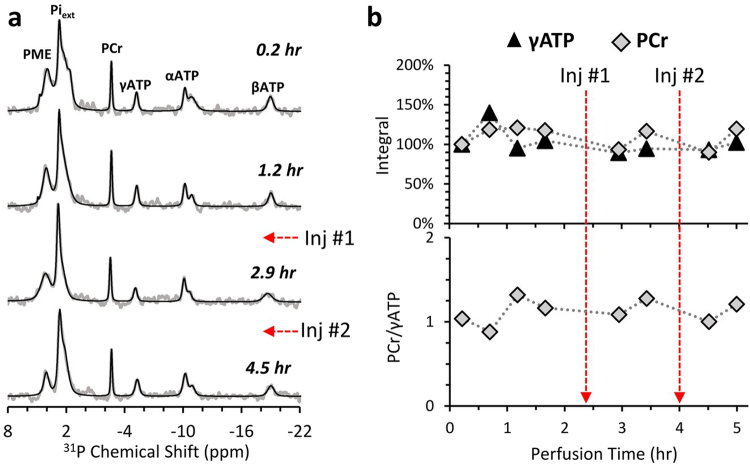
^31^P NMR monitoring of the energetic state of perfused brain slices in a set-up for hyperpolarized metabolic studies. (**a**) Typical ^31^P NMR spectra (grey) and their respective fits (black) acquired from viable rat brain slices continuously perfused in the NMR. The starting time of the acquisition of each spectrum is marked. The spectra were acquired with a 51° nutation angle, a repetition time of 3.8 s, and 8,192 points. ATP - adenosine triphosphate, PCr – phosphocreatine, Pi_ext_ - extracellular inorganic phosphate, PME – phosphomonoesters. Each spectrum is the sum of two 25.3 min acquisitions (800 excitations in total). The spectra were processed with a line-broadening of 15 Hz and zero-filled to 16,384 points. The baseline was corrected such that the wide phospholipid signal was removed. The spectra were then fit using DMFIT^44^. (**b**) Changes in the intensity of the γATP and PCr peaks relative to the first ^31^P spectrum (upper panel) and in the ratio between these two peaks (lower panel). The times of two injections of 14 mM of hyperpolarized [1-^13^C]pyruvate are indicated with red arrows.

We were able to prepare and maintain the viability of rat brain slices for more than five hours under controlled perfusion conditions, designed to ensure adequate oxygen and nutrient supply, and controlled temperature. To monitor the viability status of the slices within the spectrometer, in a non-destructive way, we used ^31^P NMR. Typical ^31^P NMR spectra are presented in Fig. 1. The signals observed include the α, β, and γ phosphate ^31^P nuclei of adenosine triphosphate (ATP), the ^31^P nucleus of phosphocreatine (PCr), inorganic phosphate (Pi), and a small signal due to phosphomonoesters signal (PME). The Pi signal that arises from the aCSF (Pi_ex_) overlaps the intracellular Pi signal (Pi_intra_) if the intracellular pH is the same as that of the aCSF (7.4). However, if the intracellular pH acidifies, the Pi_intra_ appears to the right of the Pi_ex_ signal (at a lower field) at a chemical shift indicative of the intracellular pH^33^. Figure 1 shows such a typical ^31^P monitoring, demonstrating that the same ^31^P profile could be detected several times within a duration of 5.3 h, *i.e*. the overall level of the high energy phosphates PCr and ATP was preserved throughout the hyperpolarized experiments. After injection of hyperpolarized pyruvate solution, their ratio appears to be stable, suggesting a stable energetic state.

### Metabolic products

**Figure 2:**
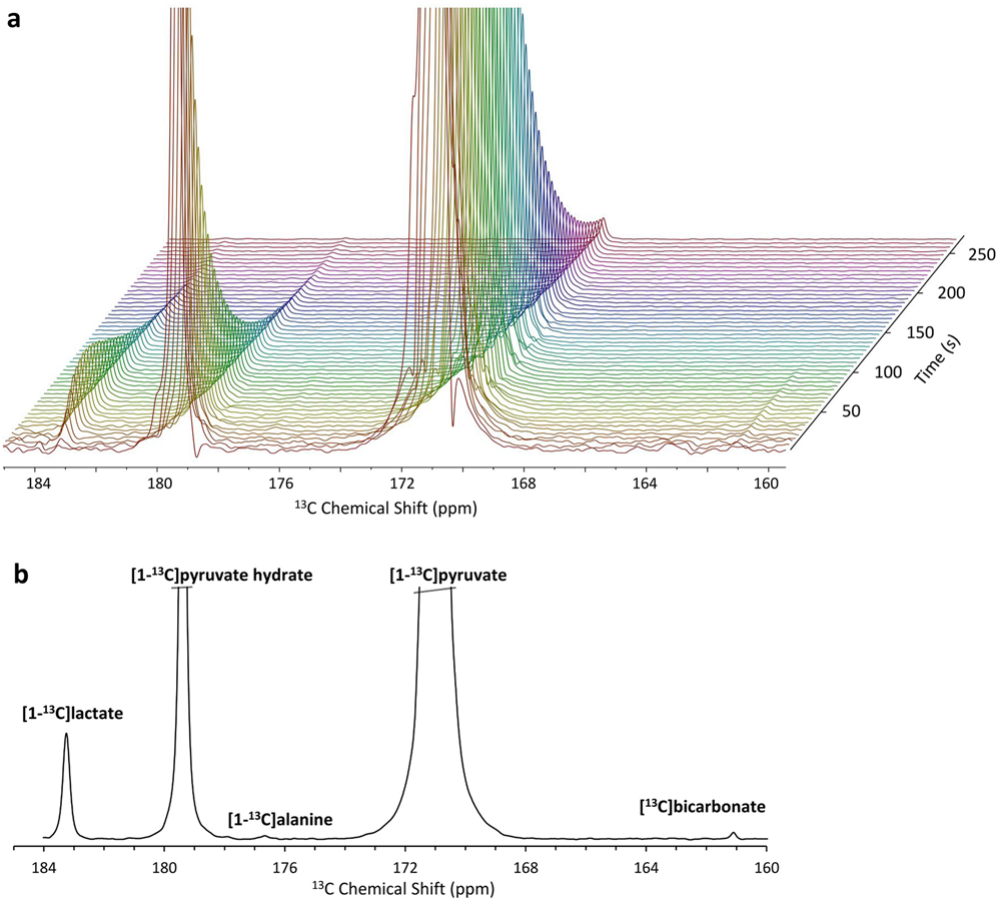
Typical ^13^C NMR spectra of brain slices in the presence of 14 mM hyperpolarized [1-^13^C]pyruvate. (**a**) Stacked ^13^C NMR spectra acquired with a 10° nutation angle and a repetition time of 5 s (10 Hz line broadening). (**b**) A ^13^C NMR spectrum showing the sum of 12 spectra acquired between 15 and 70 s from the start of dissolution. In both panels, the signals of [1-^13^C]pyruvate and [1-^13^C]pyruvate hydrate are truncated to allow dynamic range for visibility of the metabolite signals.

Upon injection of hyperpolarized [1-^13^C]pyruvate, we were able to clearly observe the appearance of two hyperpolarized metabolic products: [1-^13^C]lactate and [^13^C]bicarbonate. These signals are demonstrated in Fig. 2, and their build-up and decay can be observed at 183.3 and 161.1 ppm, respectively. The decay of the signals of [1-^13^C]pyruvate and its hydrate form can be observed at 171.0 and 179.4 ppm, respectively; both signals originate from the hyperpolarized media and are truncated to enable sufficient dynamic range to observe the changes in the signals of the metabolic products. Figure 2b demonstrates a sum of 12 spectra, acquired between 15 and 70 s from the start of dissolution in which the metabolite signals are observed with better signal-to-noise ratio.

This result unambiguously demonstrates the ability of the perfused brain tissue slices to utilize pyruvate within seconds of its arrival. The enzyme activities demonstrated by the appearance of [1-^13^C]lactate and [^13^C]bicarbonate are those of lactate dehydrogenase and pyruvate dehydrogenase, respectively, with the former appearing in higher activity. In some experiments a very small signal of [1-^13^C]alanine was observed as well (Fig. 2b) but in no experiments did we observe the formation of [1-^13^C]oxaloacetate. Therefore, these results do not inform on the activity of alanine transaminase or pyruvate carboxylase, respectively. This is in agreement with previous reports in rat brain that found that the activity of alanine transaminase is ~50 fold lower than that of lactate dehydrogenase^34^ and that the activity of pyruvate carboxylase in the brain is 6-18 fold lower than that of pyruvate dehydrogenase^25^. Therefore, in the current experimental conditions it could be expected that only in measurements with exceptional signal-to-noise ratio will [1-^13^C]alanine be observed and in all measurements [1-^13^C]oxaloacetate will be below the detection threshold.

### Fitting the observed signal to a kinetic model

To prevent mechanical damage to the slices during the forceful hyperpolarized solution injection, the slices were not fixed firmly in their position in the NMR tube. As a result, during the quick injection of the hyperpolarized media the slices may rise in the NMR tube (above the detection volume of the probe) and then settle back into the bottom of the NMR tube, a complication not previously experienced in bioreactors, where the living cells are immobilized and their position is fixed, or cell suspensions, where the living cells are uniformly mixed with the hyperpolarized solution and there will not be settling in the several minutes of the hyperpolarized acquisition. It is important to be able to discriminate which time points are strongly influenced by tissue settling and which time points correspond to a near static sample and can be reliably fit by the a kinetic model. However, visual examination of the slices in the tube during the experiment is impossible and examination at the end of the experiment is not sufficient to determine if there was significant sample motion during the acquisition.

We devised a method in which by inspecting the [1-^13^C]pyruvate signal alone we can monitor the dynamics of tissue displacement and settling for each injection of hyperpolarized solution.

The basic principle is this: after the initial rise while the pyruvate solution is injected, the signal of pyruvate is expected to decay at a constant rate determined by its T_1_ and the effect of repeated RF excitations. As the [1-^13^C]pyruvate is in great excess, the effects of metabolism on the pyruvate signal decay are negligible. However, if the slices were raised in the tube during the injection, then the early hyperpolarized recordings reflect less slices and more medium in the probe and as the slices settle back at the bottom of the NMR tube, the volume occupied by the slices increases and hence the medium volume decreases. Since the [1-^13^C]pyruvate is primarily located in the medium, as the partial volume occupied by the tissue slices in the NMR coil increases, during the settling process, the [1-^13^C]pyruvate signal will reflect the kinetics of this settling. The extent of the settling, therefore, can be determined from the deviation of the observed [1-^13^C]pyruvate signal from its expected signal if there was no tissue settling, as determined by fitting later time points when the effect of settling is minimal.

**Figure 3:**
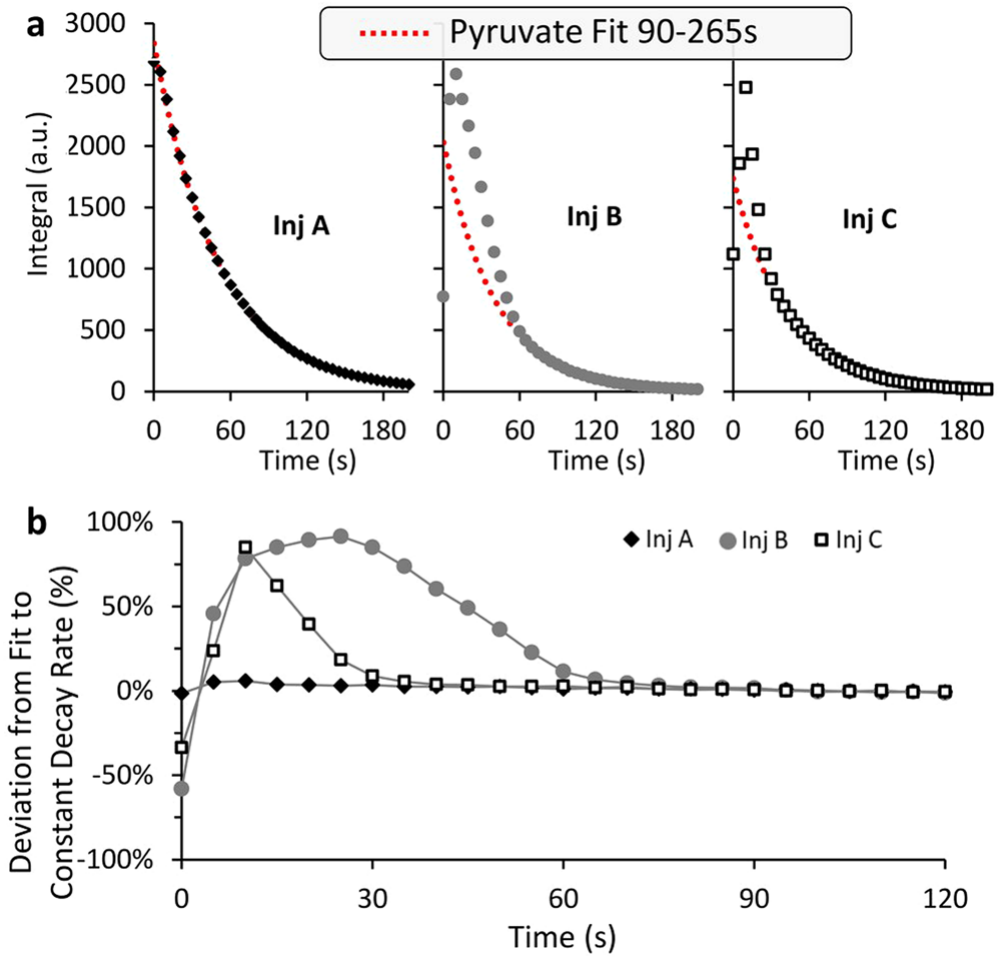
An analysis of the hyperpolarized [1-^13^C]pyruvate signal decay to determine the extent of brain slices displacement during the hyperpolarized recording. (**a**) [1-^13^C]pyruvate signal shown for three injections. Left- An injection demonstrating a near constant decay rate of the [1-^13^C]pyruvate signal; Middle- An injection in which the time points until ~60 s clearly deviate from the constant decay rate of the [1-^13^C]pyruvate signal; Right- An injection in which the time points until ~20 s deviate from the constant decay rate of the [1-^13^C]pyruvate signal. (**b**) An analysis to determine the percent deviation of the [1-^13^C]pyruvate signal from the signal expected for a constant decay rate. The constant decay rate of each experiment was determined using the time points of t >90 s. Each data point is presented as its percent deviation from the value expected for this time in the particular experiment.

Figure 3 shows three examples of hyperpolarized [1-^13^C]pyruvate signal observed for different injections evaluated by this method: in one injection (Fig. 3a, marked Inj A) one could observe that the rate of the decay of the [1-^13^C]pyruvate signal, as fit for 90–265 s, is constant throughout, suggesting that the composition of the detected volume (slices *vs*. medium) had not changed during the measurement. In another injection (Fig. 3a, marked Inj B) one could observe that the hyperpolarized signal is much higher than what is predicted by fitting later time points, suggesting significant settling until ~60 s (the initial increase in the [1-^13^C]pyruvate signal reflects the slow injection of the hyperpolarized media). In a third injection (Fig. 3a, marked Inj C), a case which is in between the first and second cases can be seen: only for the early time points, up to ~20 s, does the pyruvate signal deviate from the expected decay. This analysis can be more clearly understood when the deviation of the observed pyruvate signal from the extrapolated fit is plotted (Fig. 3b). For determining the T_1_ of pyruvate and its initial polarization, it was most accurate to fit the signals from 90 s onwards. However using this time range for the metabolic products was not possible, as an accurate determination of both the rate and the T_1_ requires including the buildup portion. Therefore, for the kinetic analysis of the hyperpolarized products, we chose a threshold of 25% deviation from the expected pyruvate signal as a time frame for when the effect of settling was minimal, and the signal of the metabolic products could be reliably fit. Of the 17 injections included in this study, for 8/17 this threshold was reached within 20 s and for 13/17 it was reached within 30 s (see Table 1).

**Table 1:** Summary of parameters from kinetic model fits.

Inj. No.	Animal No.	Conc. Pyr. (mM)	TR (s)	Pulse (°)	Pyr. Dev. ≤25% (s)	T_1,pyr_ (s)	R^2^_pyr_	T_1,lac_ (s)	t_shift_ (s)	v_Lac_ (nmole/s)	R^2^_lac_	T_1,bic_ (s)	v_Bic_ (nmole/s)	R^2^_bic_
1	Rat 1^a^	31.2	5	10	20	50.8	1.00	24.7	11.0	10.3	1.00	27.0	0.6	0.81
2		28.0	5	10	25	51.0	1.00	34.8	12.4	6.1	1.00	15.2	0.5	0.87
3	Rat 2^a^	14.3	5	10	25	54.4	1.00	19.2	20.4	11.5	0.99	*n.a*.	*n.a*.	*n.a*.
4		15.4	5	10	35	59.3	1.00	25.9	32.1	2.4	0.97	*n.a*.	*n.a*.	*n.a*.
5	Rat 3^a^	15.0	5	10	25	49.9	1.00	30.1	12.2	7.5	1.00	12.8	0.6	0.91
6		15.5	5	10	20	52.2	1.00	29.1	14.7	8.3	1.00	11.4	0.5	0.84
7	Rat 4^a^	13.9	5	10	75	45.7	1.00	27.7	50.8	7.0	0.99	*n.a*.	*n.a*.	*n.a*.
8	Rat 5^a^	14.9	5	10	55	48.2	1.00	18.3	38.7	16.1	1.00	8.5	1.1	0.87
9	Rat 6^a^	15.5	5	10	45	54.3	1.00	25.5	29.6	10.9	1.00	15.1	0.4	0.84
10	Rat 7^a^	14.3	5	10	30	56.9	1.00	28.6	11.9	7.9	1.00	16.7	0.5	0.86
11	Rat 8^a^	14.2	5	10	20	60.7	1.00	28.4	8.9	6.9	1.00	20.2	0.2	0.8
12	Rat 9^a^	13.5	5	10	20	56.6	1.00	34.9	8.9	7.3	0.99	25.9	0.5	0.85
13	Rat 10^a^	15.4	5	10	5	61.7	1.00	30.4	0.2	1.3	0.98	*n.a*.	*n.a*.	*n.a*.
14	Rat 11^a^	14.5	5	10	0	64.4	0.99	*n.a*.	*n.a*.	*n.a*.	*n.a*.	*n.a*.	*n.a*.	*n.a*.
15		14.4	5	10	5	70.0	0.98	*n.a*.	*n.a*.	*n.a*.	*n.a*.	*n.a*.	*n.a*.	*n.a*.
16	Rat 12^b^	15.4	5	20	40	73.2	1.00	18.7	27.3	20.8	1.00	9.9	2.0	0.93
17	Rat 13^b^	16.4	5	15	10	67.8	1.00	49.3	3.2	7.8	0.95	*n.a*.	*n.a*.	*n.a*.

^a^Anesthetized with ketamine/xylazine, ^b^Anesthetized with isofluorane.

*n.a*. –rate could not be determined due to low signal intensity.

Conc. Pyr. – The actual concentration of [1-^13^C]pyruvate in the dissolution.

Pyr. Dev. – Deviation of the pyruvate signal decay from the constant decay rate

TR – Repetition time.

Inj. No. – the number of hyperpolarized [1-^13^C]pyruvate injection in the order in which it was actually performed.

**Figure 4:**
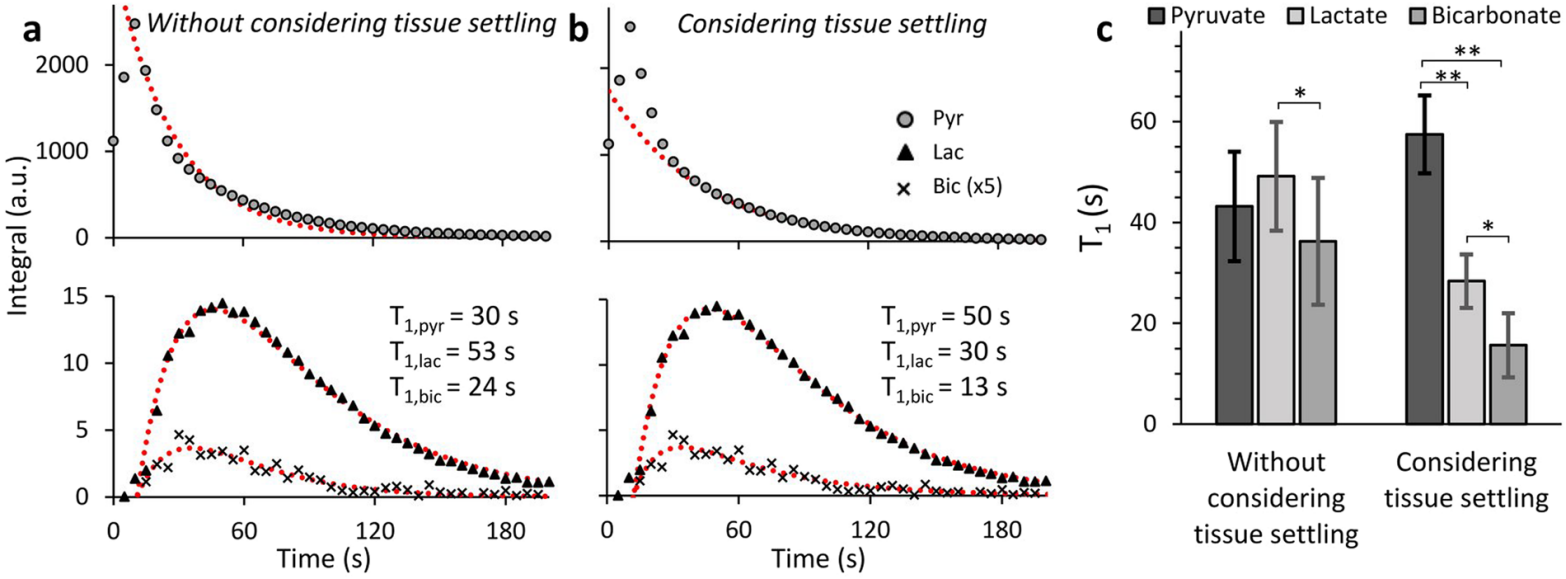
Kinetic and relaxation rate constants after administration of 15.0 mM hyperpolarized [1-^13^C]pyruvate to viable rat brain slices.(**a**) The integrated intensities of [1-^13^C]pyruvate (Pyr) (●) [1-^13^C]lactate (Lac) (▲), and [^13^C]bicarbonate (Bic) (**x**) and their fit to the kinetic model (dashed lines). Tissue settling was not considered and therefore all time points from the completion of the injection, *i.e*. from 10 s onwards were fit. Fitting this time range yielded T_1,pyr_ = 30 s, T_1,lac_ = 53 s and T_1,bic_ = 24 s. Assuming that 0.5 ml of the volume detected by the coil is occupied by 15 mM [1-^13^C]pyruvate solution (and about 0.9 ml are occupied by the tissue) metabolic rates of v_lac_ = 4.2 nmole/g/s and v_bic_ = 0.3 nmole/g/s can be determined (R^2^_pyr_ = 0.98, R^2^_lac_ = 0.99, R^2^_bic_ = 0.88). (**b**) The integrated intensities of the same signals and their fit to the kinetic model (dashed lines) taking into consideration the tissue settling process. In order to minimize the effect of potential tissue displacement on the resultant parameters, the [1-^13^C]pyruvate signal was fit from 90 s onwards, while the [1-^13^C]lactate and [^13^C]bicarbonate signal were fit from when the deviation of the pyruvate signal from the constant decay was 25% or less, in this case from 25 s onwards. Fitting this time range yielded T_1,pyr_ = 50 s, T_1,lac_ = 30 s and T_1,bic_ = 13 s. Using the same assumptions as above, the metabolic rates were v_lac_ = 7.5 nmole/g/s and v_bic_ = 0.6 nmole/g/s. (R^2^_pyr_ = 1.00, R^2^_lac_ = 1.00, R^2^_bic_ = 0.91). We note that the mismatch between the early experimental data points of [1-^13^C]pyruvate and the model curve likely reflects tissue displacement in the beginning of the experiment. Tissue displacement may also explain the slight mismatch in the early [1-^13^C]lactate and [^13^C]bicarbonate time points. (**c**) Longitudinal relaxation time constants for the 17 injections that were analyzed. For 2 injections severely reduced SNR prevented fitting the lactate signal and for 4 additional experiments SNR was not sufficient to fit the bicarbonate signal. Without considering tissue settling we found the following T_1_ parameters: T_1,pyr_ = 43 ± 11 s (n = 17), T_1,lac_ = 49 ± 11 s (n = 15) and T_1,bic_ = 36 ± 3 s (n = 11). When tissue settling was considered in the manner described above the following T_1_ parameters were determined: T_1,pyr_ = 57 ± 8 s (n = 17), T_1,lac_ = 28 ± 5 s (n = 15) and T_1,bic_ = 16 ± 6 s (n = 11). *p < 0.001, **p < 0.0001.

The fit obtained when the effect of tissue settling is not considered and when it is considered shows the importance of including this effect when fitting the data to a kinetic model. This difference is demonstrated for a single dataset that is fit without considering tissue settling (Fig. 4a) and with considering tissue settling (Fig. 4b). For this dataset, it can be seen that when tissue settling is not considered, and all time points from the completion of the injection are included in the fit, the [1-^13^C]pyruvate signal is poorly fit (Fig. 4a, upper panel), and the resulting fit T_1_ is much shorter than expected for [1-^13^C]pyruvate in aCSF. Furthermore, although the fit of the [1-^13^C]lactate and [^13^C]bicarbonate signal appears good, it yields a T_1_ of lactate that is longer than the T_1_ of pyruvate, the opposite of what is predicted by *in situ* measurements^35^ (Fig. 4a). On the other hand, when tissue settling is considered- by fitting pyruvate from 90 s onwards and by fitting the metabolic product signals from time points with less than 25% deviation of the pyruvate signal from the fit- the time course of all metabolites is well fitted (Fig. 4b). Additionally, the fit T_1_ value of [1-^13^C]pyruvate is longer than that of [1-^13^C]lactate in accordance with what is predicted by *in situ* measurements^35^.

When the T_1_s obtained for all datasets fit to the kinetic model with and without considering tissue settling are analyzed, the importance of considering tissue settling can be clearly seen: When tissue settling is not considered there is large variability in the fit T_1_ parameters and no significant difference between the T_1_ of the different metabolic products and pyruvate (Fig. 4c). By contrast, when tissue settling is considered in the manner described above, the variability in the T_1_ values for the different metabolites is much smaller, and significant differences can be observed between the T_1_s of the different metabolites (Fig. 4c). In our system we find for [1-^13^C]pyruvate a T_1_ of 57 ± 8 s (n = 17), for [1-^13^C]lactate a T_1_ of 28 ± 5 s (n = 15), and for bicarbonate a T_1_ of 16 ± 6 s (n = 11).

The signal of hyperpolarized pyruvate can be used as an internal standard as its concentration is known. To obtain metabolic rates we assume that after settling 0.5 ml of hyperpolarized solution is observed in the coil, while the remaining volume of approximately 0.9 ml is occupied by 0.9 g brain tissue slices.

Using this analysis and assumption, the rate of lactate production after addition of 14 ± 2 mM of hyperpolarized [1-^13^C]pyruvate was determined to be 8.9 ± 5.2 nmole/g/s (n = 13) and the rate of bicarbonate production was determined to be 0.8 ± 0.6 nmole/g/s (n = 9) (Table 1). The large variation in the fit metabolic rates most likely reflects changes in the amount of viable cells in the brain slices or changes in packing of the slices during the measurement which will affect both the amount of slices whose metabolism is observed as well as the volume free for the hyperpolarized pyruvate solution. In the future, we aim at improving the quantification of these two experimental variables in order to be able to determine absolute metabolic rates.

When we compared the metabolic rates obtained using this model after injection of 14 ± 2 mM of [1-^13^C]pyruvate with the product area-under-the-curve (AUC) normalized by the pyruvate AUC, we observed no correlation between the normalized lactate AUC to v_lac_ (R = 0.313, n = 13) or between the normalized bicarbonate AUC to v_bic_ (R = 0.039, n = 9).

Similar metabolic rates were determined when 28 ± 2 mM of hyperpolarized [1-^13^C]pyruvate was added to the slices: the rate of lactate production was determined to be 8.2 ± 3.0 nmole/g/s (n = 2) and bicarbonate production was determined to be 0.5 ± 0.1 nmole/g/s (n = 2). This result reinforces that a zero order kinetic model is appropriate in this system because the substrate is already in excess at 14 mM. The rate of metabolite production is likely limited by the amount of pyruvate entering the brain cells, which is already maximal at 14 mM.

**Figure 5:**
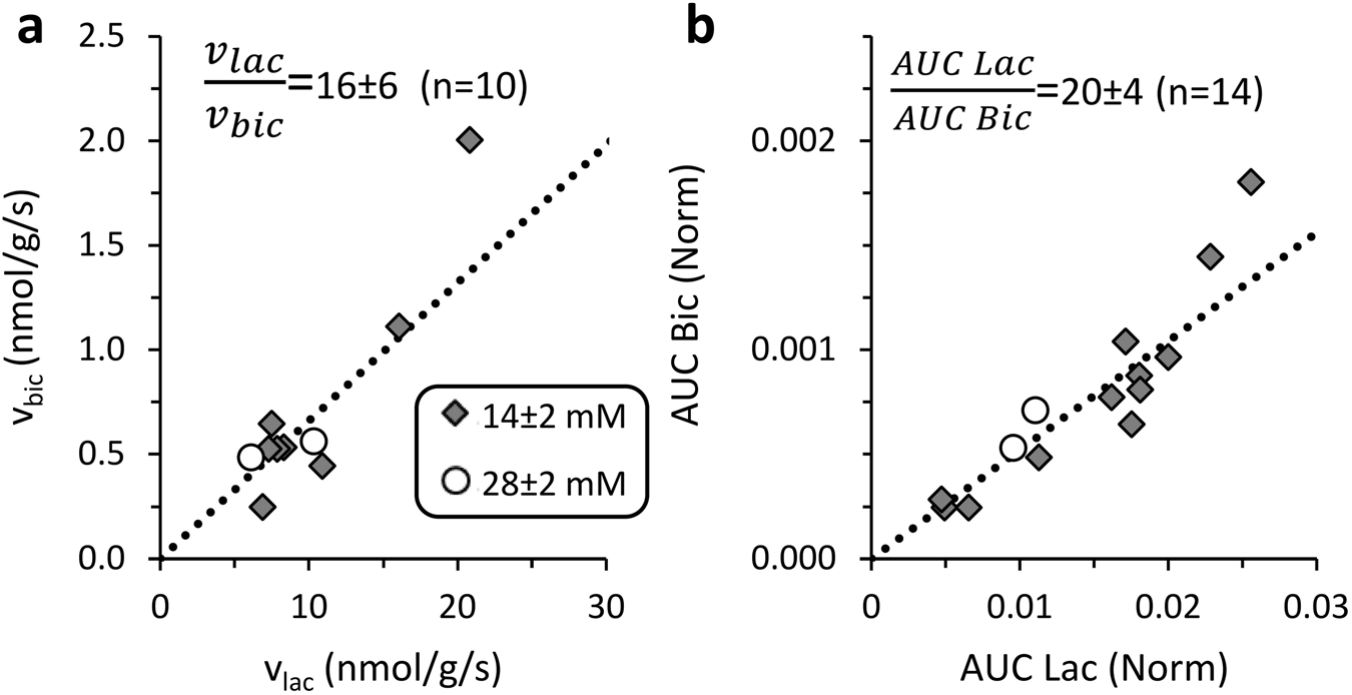
Correlation between [1-^13^C]lactate and [^13^C]bicarbonate production. (**a**) Correlation between the rates of bicarbonate and lactate production as determined with the kinetic model after injections of 14 ± 2 mM (grey diamonds) or of 28 ± 2 mM (open circles) of [1-^13^C]pyruvate. The dotted line corresponds to the average ratio of lactate to bicarbonate production, 16 ± 6 (n = 11). (**b**) Correlation between the normalized bicarbonate and lactate AUCs for the same injections of 14 ± 2 mM (grey diamonds) or of 28 ± 2 mM (open circles) of [1-^13^C]pyruvate. The dotted line corresponds to the average ratio of lactate to bicarbonate AUC, 20 ± 4 (n = 14).

However, despite the large variation in the calculated metabolic rates, we observed a strong correlation between the rates of lactate and bicarbonate production (R = 0.924, n = 10). The ratio of v_lac_ to v_bic_ was found to be 16 ± 6 (n = 10) (Fig. 5a), corresponding to a 16-fold higher rate of lactate production. Furthermore, although the normalized lactate and bicarbonate AUCs did not correlate with their respective metabolic rates, there was a strong correlation between these two parameters for each injection (R = 0.922, n = 14); the ratio of the lactate AUC to bicarbonate AUC was found to be 20 ± 4 (n = 14) (Fig. 5b). This ratio is significantly larger than the v_lac_ to v_bic_ ratio (p = 0.036), however in light of the results of the kinetic model this can be attributed to the shorter bicarbonate T_1_ (see Fig. 4) which causes its signal to accumulate to a lesser degree, skewing the total signal ratio to higher values.

A third analysis tool was also applied to the data. In this tool, developed previously in our lab^36^, the data were fit to a first order kinetic model and the rate constants for the metabolic productions are calculated. The same time frames for each injection were analyzed as in the zero order kinetic model, *i.e*. tissue settling was taken into consideration. For the injections with 14 ± 2 mM of [1-^13^C]pyruvate the k_lac_ was 10.5 ± 5.1 × 10^−4^ s^−1^ (n = 12) and the k_bic_ was 1.5 ± 0.7 × 10^−4^ s^−1^ (n = 8).

## Discussion

We demonstrate a method to characterize the metabolism of hyperpolarized substrates in viable brain tissue. By utilizing perfused brain slices we maintain the cellular heterogeneity and microstructure of the brain but eliminate the convoluting effects of (1) influx of metabolites produced in other organs to the brain, (2) the rate of BBB transport (3) efflux of metabolites produced in the brain, and (4) MRS voxel contamination by surrounding tissues and blood vessels. In this way, we are able to rapidly characterize the metabolic characteristics of brain tissue in a setup where the microenvironment can be controlled and finely tuned. Specifically with regard to the potential influx of hyperpolarized metabolites from the periphery, we note that the rat circulation time is fast (less than 5 s), while metabolite signals in rodent tissues investigated *in vivo* by hyperpolarized MRS last for more than 1 min, regardless of the route of administration (intra-arterial or intravenous)^37^. Thus, it is highly likely that *in vivo* some degree of influx of metabolites to the brain from the periphery exist. For this reason, a study on the isolated brain is important.

Multiple studies have been published studying hyperpolarized metabolism in perfused whole organs^38,39^. To the best of our knowledge this is the first description of hyperpolarized NMR investigation of metabolism in brain slices. Unlike the case of perfused organs, in the current system the tissues can move in the tube. In order to fit the hyperpolarized metabolic data with a simple model it must be ascertained that the rapid injection necessary for hyperpolarized measurements does not cause significant tissue motion. However, from time to time, due to slight changes in injection speed, pressure is produced at the bottom of the tube causing the slices to move upwards (and then settle back down). Attempts to limit this movement failed and led to slice tear when compressed against the limiting object (porous filters). To deal with the occasional slice movement and still obtain useful kinetic data we described a simple methodology to inspect in retrospect the motion of tissue samples and determine the time points that can be accurately used to fit with the kinetic model.

Previous studies have shown the difficulty of interpreting metabolic rates from *in vivo* hyperpolarized spectroscopy of the brain. Marjanska *et al*.^16^ found that the observed ratios of pyruvate to its products in the brain, *in vivo*, strongly depended on the flip angle used, and they attributed this result to increased contribution from other tissues and blood vessels when lower flip angles were used, an effect known as MRS voxel contamination in the *in vivo* MRS jargon. This effect is obviously avoided when using *ex-vivo* brain preparations such as the one used here. Josan *et al*.^13^ demonstrated that the level of isoflurane anesthesia affected the observed product to pyruvate ratios, presumably due to the vasodilating effect of increasing doses of isoflurane, which increased the contribution of blood vessels to the observed voxels. Further, in order to characterize the intrinsic metabolic rates of the brain, it was necessary to simultaneously measure the concentration of hyperpolarized metabolites in the blood and input this information into a complex model that accounted for the contribution of blood vessels to the observed signal as well as the rate of blood brain barrier transport^18^.

In the literature there is some ambiguity regarding the conversion of hyperpolarized [1-^13^C]pyruvate to [1-^13^C]alanine in brain tissue. In some studies^12,16^ alanine has been observed in the brain tissue. On the other hand, Mayer *et al*.^15^ showed that when high resolution spectroscopic imaging is used there is no observable alanine signal in the brain, suggesting that when hyperpolarized alanine is observed in the brain it is due to contamination of brain MRS voxels with other tissues. The minute amount of hyperpolarized alanine in our brain slice model reinforces the claim of Mayer *et al*.^15^ that the hyperpolarized alanine observed *in vivo* does not originate predominantly in the brain tissue. Interestingly, Chavarria *et al*.^12^ observed an increase in the cerebral alanine-to-pyruvate ratio in the case of acute liver failure and it would be worthwhile to reproduce this result in a brain slices model, in order to understand the phenomena that they observed.

Taken together, previous studies of hyperpolarized pyruvate metabolism in the brain suggest a bicarbonate to lactate ratio of (1:5-30)^12,13,15,16,19,20^. We note that for five of the six studies aimed at this characterization (DeVience *et al*.^19^, Guglielmetti *et al*.^20^, Chavarria *et al*.^12^, Mayer *et al*.^15^, and Josan *et al*.^13^) this reported ratio relates to the intensity ratios of the bicarbonate and the lactate signals. Only in the study of Marjanska *et al*.^16^ the time courses of metabolite signals were modeled and the rates of production were calculated. In that study, the signal intensity ratios were similar to the other 5 studies with lactate much higher than bicarbonate for all time points, although the calculated rate of production was only slightly higher for lactate. In principle, many sources of variability for this ratio exist in *in vivo* studies due to the use of different external magnetic fields, different RF coils with different localization properties, and different excitation RF pulses. These factors could influence the relative signals and contribution of metabolites flowing into the detected region from other regions in the body. In addition, variation in T_1_ of the metabolites which depends on their location (extracellular or intracellular)^40^ and field strength could alter the results and the interpretation of the *in vivo* studies. For this reason, it is likely that signal intensity ratios on hyperpolarized experiments will vary across laboratories due to the obligatory variation in external magnetic fields, RF probes, pulse sequences, and intravenous administration protocols. However, enzymatic rates calculated with proper kinetic models should be reproducible. Due to the many sources of variability on *in vivo* hyperpolarized MR experiments and the complexity of modeling the data, as demonstrated by Marjanska *et al*.^16^, it appeared reasonable to compare our results to data obtained by well established biochemical assays which were in the same range as determined here in real time (see results). The ratio of production rates of [^13^C]bicarbonate to [1-^13^C]lactate that we have observed, approximately 1:16, corresponds with the analysis of signal intensity ratios which was carried out here as well (Fig. 5) and appears with the same trend which was previously observed for the signal intensity ratios *in vivo*^12,13,15,16,19,20^.

Unlike the lactate-to-pyruvate and bicarbonate-to-pyruvate ratio that strongly depend on the volume of viable tissue, *ex vivo*, and blood vessel volume and BBB transport *in vivo*, we believe that the magnitude of the bicarbonate-to-lactate production rates ratio may be a marker of brain tissue status. This conjecture is supported by the recent studies that showed that the corresponding signal intensity ratio is significantly decreased in an animal model of TBI compared to the contralateral unaffected hemisphere^19,20^, and there is a smaller but still significant reduction in this ratio in the case of injury that is not TBI^19^. Notably, the cerebral lactate-to-pyruvate ratio (LPR), clinically measured by cerebral microdialysis, has been shown to be an important indicator in the clinical case of TBI, as a high LPR can be associated with a poor outcome based on the extent and time course of its elevation^41,42^ and a decrease in the LPR index may be an indicator of therapeutic efficacy^43^.

Further studies on the relationship between pyruvate metabolism and brain microenvironment conditions and diseases are now warranted using the system developed here, as well as research into other potential brain metabolic probes.

## Methods

### Chemicals

The OXO63 radical (GE Healthcare, UK) was obtained from Oxford Instruments Molecular Biotools (Oxford, UK). [1-^13^C]pyruvic acid was purchased from Sigma-Aldrich, (Rehovot, Israel) and from Cambridge Isotope Laboratories (Tewksbury, MA, USA). NaCl, KCl, D-glucose, NaHCO_3_, MgCl_2_, NaH_2_PO_4_(2H_2_O), and CaCl_2_ were purchased from Sigma-Aldrich, (Rehovot, Israel). Ketamine solution (100 mg/ml), xylazine solution (23.32 mg/ml), and isoflurane were obtained from the Institutional Authority for Biological and Biomedical Models.

### Artificial cerebrospinal fluid (aCSF) and the perfusion system

The aCSF used for slice perfusion contained 125 mM NaCl, 2.5 mM KCl, 15 mM D-glucose, 26 mM NaHCO_3_, 1 mM MgCl_2_, 1.25 mM NaH_2_PO_4_, and 2 mM CaCl_2_ in water (90/10 v/v double-distilled H_2_O/D_2_O). The medium was bubbled with 95%/5% O_2_/CO_2_ for 1 hour prior to slice perfusion and continuously bubbled with this gas mixture throughout the experiment at a flow rate 0.4 l/min. The pH of the medium was 7.4. A 200 ml reservoir of this aCSF was kept in a water bath at 40 °C outside the NMR spectrometer and was delivered to the NMR tube *via* medical grade extension tubes and pumped in a closed circle with a peristaltic pump (Masterflex L/S Analog Pump Systems, Cole-Parmer, IL, USA). Thin polyether ether ketone (PEEK) lines (i.d. 0.040′′, Upchurch Scientific, Inc., Oak Harbor, WA, USA) were used for in- and out-flow of the aCSF or hyperpolarized media to and from the brain slices located in the confined space at the bottom of the 10 mm NMR tube inside the spectrometer bore. The magnetic susceptibility of PEEK is similar to water and therefore can be used during NMR spectroscopy recordings. The temperature of the slices during the perfusion inside the NMR spectrometer was 32 °C. The temperature was calibrated independently with a thermocouple prior to each experiment.

### Animals

Female Sprague-Dawley rats (n = 12, 3–5 months old, 140–170 g) were obtained from the Institutional Authority of Biological and Biomedical Models. All the experimental procedures were in accord with the regulations of the Institutional Animal Care and Use Committee of the Hebrew University. Care was taken to minimize pain and discomfort to the animals. Anesthesia of 11 of the 13 animals was performed with an *i.p*. injection of ketamine and xylazine mixture (0.85:0.15 v/v) at a dose of 0.12 ml mixture per 100 g body weight. Two animals were anesthetized with isoflurane using a gas anesthesia system (Somnosuite, Kent Scientific, Torrington, CT, USA). For induction we used 3.5% isoflurane and 440 ml/min of room air. For maintaining anesthesia, we used 2.8–3.0% isoflurane and the same air flow.

The brains of 13 animals were used here in total. Each brain slices sample was produced from one animal. Altogether, 17 experiments are described herein, which were performed on 13 brain slices samples. On 4 of these brain slices samples we performed two injections of hyperpolarized [1-^13^C]pyruvate.

### Surgical procedure and slice preparation

**Figure 6:**
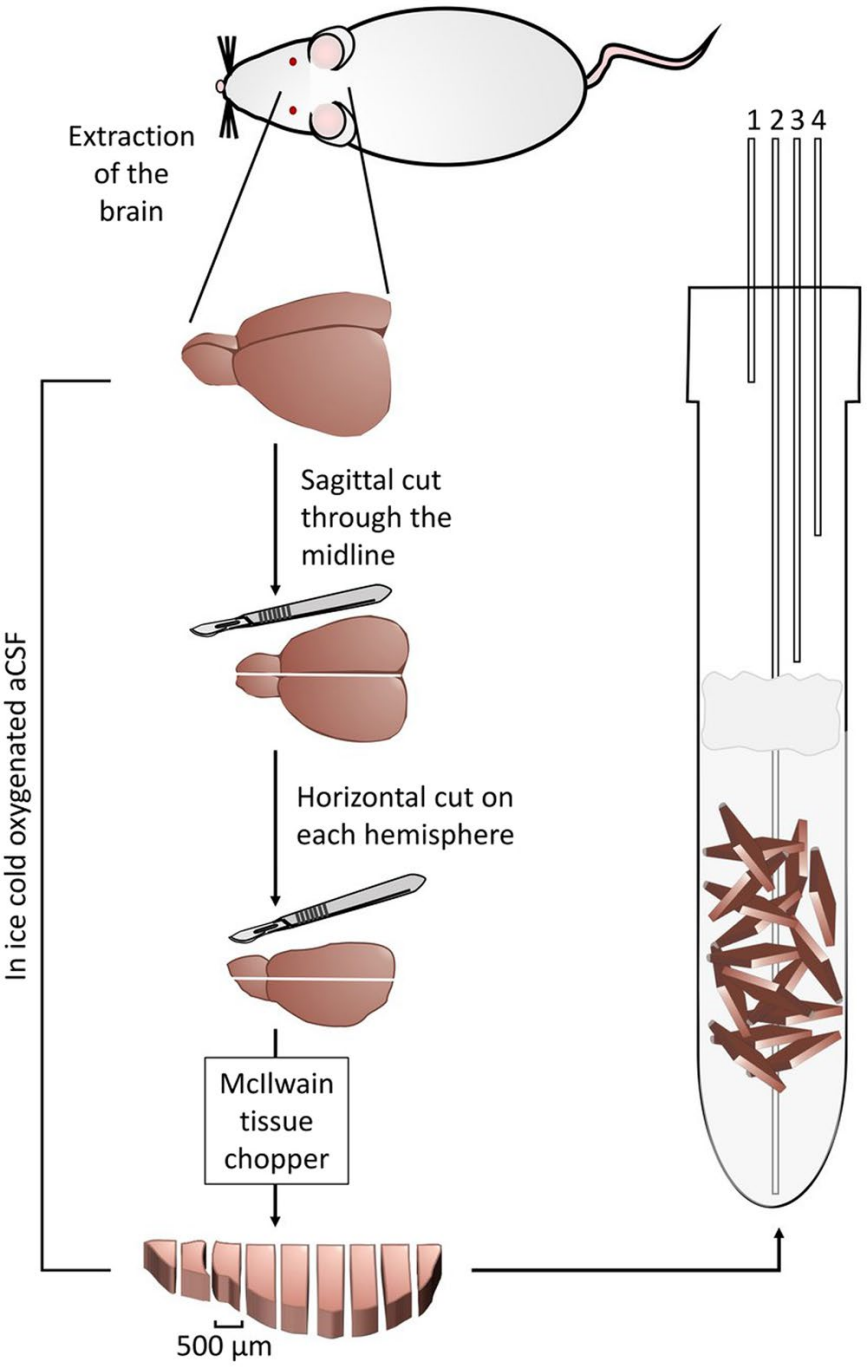
Obtaining rat brain slices and maintaining their viability in an NMR tube. The brain is extracted from an anesthetized rat and placed in ice-cold aCSF. Then the brain is cut into four segments which are kept in ice-cold aCSF. Then each segment is sliced to 500 μm slices which are placed back into ice-cold aCSF. When all segments have been sliced, the slices are transferred to a 10 mm NMR tube and are perfused therein with the same aCSF at 37 °C. The lines going in and out of the NMR tube are marked as follows: (1) Humidified 95%/5% O_2_/CO_2_ atmosphere; (2) Inflow line, carrying medium from an external 200 ml aCSF pool to the NMR tube. The external medium pool (not shown here) is kept at 40 °C and is continuously bubbled with humidified 95%/5% O_2_/CO_2_. This line is also used for the hyperpolarized pyruvate injection; (3) Out-flow line, carrying aCSF from the NMR tube to the external pool; (4) Backup outflow line. The slices are not fixed in the NMR tube but a filter made of cotton balls (colored gray in the figure) is placed a few cm above the slices to prevent suction of the occasional floating slice into the out-flow lines. The slices are kept at the bottom of the tube, at the region visible to the NMR probe, due to gravity and possible inter slice adhesion and the gentle perfusion generally does not displace them.

The surgical procedure began after obtaining a negative pedal pain reflex. First, the diaphragm was exposed by a subcostal incision. Then, it was removed to allow view of the heart. The right atrium was then cut to allow drainage of blood and 30 ml of ice-cold aCSF were injected into the left ventricle over *ca*. 5 min, taking care not to allow the passage of air bubbles to the heart and circulation. This procedure was done to wash blood away from the brain prior to the brain extraction and slicing. The animals were then sacrificed by decapitation. The brain was rapidly removed and placed into ice cold aCSF (~1–2 °C). The cerebrum was separated from the cerebellum and whole cerebrum precision cut slices (500 µm) were prepared using a McIlwain tissue chopper (The Mickle Laboratory Engineering Company Ltd., Surrey, UK). The process of brain extraction and slicing the entire cerebrum took about 30 min from the moment of decapitation. Throughout this time the tissue was constantly kept in the ice cold aCSF (See Fig. 6).

### DNP spin polarization and dissolution

Spin polarization and fast dissolution were carried out in a dissolution-DNP spin polarization device (HyperSense, Oxford Instruments Molecular Biotools, Oxford, UK) operating at 3.35 T. Microwave frequency of 94.110 GHz was applied for the polarization of a [1-^13^C]pyruvic acid formulation at 1.40 to 1.49 K. The formulations consisted of 11.1 to 14.0 mM OX063 radical in the neat acid. The amount of [1-^13^C]pyruvic acid formulation placed in the polarization cup was 5 ± 0.5 mg in 15 of the experiments described herein and 10 ± 1 mg in 2 experiments. The dissolution medium consisted of 4 ml of 50 mM phosphate buffer which contained 19 mM TRIS and 138.6 mM NaCl. The pH of the dissolution medium was adjusted with NaOH such that upon mixing with the pyruvic acid in the cup the final pH was 7.4.

### NMR Spectroscopy

^31^P and ^13^C NMR spectroscopy were performed in a 5.8 T high resolution NMR spectrometer (RS2D, Mundolsheim, France) located about 2.2 meters away from the spin-polarization magnet (center-to-center), using a 10 mm broad-band NMR probe.

^13^C spectra were acquired with a 10° (n = 15) or 15° (n = 1) or 20° (n = 1) nutation angle and a repetition time of 5 s. The bandwidth was selected to detect the entire spectral width of the ^13^C spectrum (300 ppm) enabling simultaneous detection of hyperpolarized [1-^13^C]pyruvate and its metabolites.

^31^P spectra of thermal equilibrium phosphate signals were acquired with a nutation angle of 51° and a repetition time of 3.8 s. The bandwidth used (~100 ppm) was larger than the span required to detect the high energy phosphates (~40 ppm) due to technical limitations of the instrument. This introduced about 20% increase in the noise level.

The probe was tuned back and forth from ^31^P to ^13^C during the experiment to support the requirement of the experimental workflow, in which several injections of hyperpolarized media were performed into the same perfused brain slices sample and the energy status was monitored using ^31^P spectroscopy before, in between, and after the injections. Homogeneity optimization (shim) was performed using the water signal on the ^1^H channel and using the lock system. To support the latter, the aCSF was supplemented with 10% D_2_O in all of the experiments.

### Experimental design - hyperpolarized media injection and work flow

In order to perform hyperpolarized experiments, it is important to be able to rapidly inject a small volume of hyperpolarized solution directly to the brain slices. The hyperpolarized solutions were injected *via* a Teflon line from the spin-polarizer into a conical tube placed at the fringe field of the magnet within 6 s of Helium (g) chase. Then the hyperpolarized media were manually transferred *via* a medical manifold (connected to the in-flow line and made of a combination of medical grade 3-way valves and syringes) to the NMR tube containing the brain slices, injecting the hyperpolarized media to the very bottom of the tube. Altogether, the duration of the process of transferring the hyperpolarized media was completed within 15 s from the start of the dissolution process. The greenish color of the radical solution enabled visual inspection of the resulting solution (after the measurement) and ensured the homogenous distribution of the hyperpolarized media around the brain slices in the NMR tube. The hyperpolarized media was injected gently to minimize tissue displacement and care was taken to avoid the introduction of air bubbles that could interfere with magnetic field homogeneity due to the large difference in magnetic susceptibility between air and water. The experimental work flow and setup are illustrated in Fig. 6.

Immediately prior to dissolution, the perfusion was stopped and the manifold system was rotated to allow rapid injection of the hyperpolarized solution. In this setup, perfusion was stopped ~30 s before injection of the hyperpolarized solution and was resumed only after acquisition of hyperpolarized spectra was completed (approximately 4 min). This was done in order to characterize the metabolism of a constant concentration of [1-^13^C]pyruvate without effects of wash-in and wash-out. ^13^C NMR acquisition was started immediately at the start of the dissolution process in the spin-polarizer, such that the first acquisitions show only noise and then the entrance of the hyperpolarized media into the tube and subsequent metabolism are recorded in real-time.

### Processing and Data Analysis

Spectral processing was performed using MNova (Mestrelab Research, Santiago de Compostela, Spain). Integrated intensities were calculated either with MNova or with DMFIT^44^.

### Kinetic Model

#### Zero-order reaction kinetics

We were interested in characterizing the metabolism of [1-^13^C]pyruvate in brain slices using a simple zero-order kinetic model for the metabolic processes. By comparison of the intensity of the [1-^13^C]pyruvate signal to the metabolite signal, it can be seen that the concentration of pyruvate is virtually unchanged throughout the hyperpolarized acquisition window, hence it is possible to use zero-order reaction kinetics in the analysis. The decay of the [1-^13^C]pyruvate signal is best fit by modeling the effects of its longitudinal relaxation (*T*_*1,Pyr*_) and low flip angle (*θ*) RF pulses repeated at constant intervals *TR*, neglecting the decay due to metabolism, as described by the equation below.

$${S}_{Pyr}(t)={S}_{Pyr}(0)\cdot {e}^{-\frac{t}{{T}_{1,Pyr}}}\cdot {(cos\theta )}^{\frac{t}{TR}}$$

To model the signal of [1-^13^C]lactate and [^13^C]bicarbonate we will treat each of these metabolic conversions as independent. To the best of our knowledge, there is no reason to believe otherwise. The signal behavior for each product can be described as follows: the hyperpolarized signal is building up due to its synthesis (from the hyperpolarized substrate) and at the same time the signal decays due to longitudinal relaxation of the substrate and product and repeated low flip angle excitations. We modeled the product signal using a previously developed model that takes into account the T_1_ heterogeneity (*i.e*. the T_1_s of the products and the substrate are not assumed to be equal)^45^, which defines the difference in longitudinal relaxation time constants of the product and precursor as follows:

$${\rm{\Delta }}{\rho }_{Lac}=\frac{1}{{T}_{1,Lac}}-\frac{1}{{T}_{1,Pyr}}\,{\rm{and}}\,{\rm{\Delta }}{\rho }_{Bic}=\frac{1}{{T}_{1,Bic}}-\frac{1}{{T}_{1,Pyr}}$$

Assuming that due to mixing effects metabolism does not start immediately, we introduced a time shift factor, t_shift_, to the model which reflects this time lag between the appearance of hyperpolarized pyruvate signal and the formation of metabolic products.

Using these assumptions the signals *S*_*Lac*_(*t*) and *S*_*Bic*_(*t*) can be described by the equations below:

$${S}_{Lac}(t)=\frac{{S}_{Pyr}(0)}{[Pyr](0)}\cdot \frac{{v}_{Lac}}{{\rm{\Delta }}{\rho }_{Lac}}\cdot {e}^{-\frac{(t-{t}_{shift})}{{T}_{1,Pyr}}}\cdot {(cos\theta )}^{\frac{(t-{t}_{shift})}{TR}}\cdot (1-{e}^{-(t-{t}_{shift})\cdot {\rm{\Delta }}{\rho }_{Lac}})$$

$${S}_{Bic}(t)=\frac{{S}_{Pyr}(0)}{[Pyr](0)}\cdot \frac{{v}_{Bic}}{{\rm{\Delta }}{\rho }_{Bic}}\cdot {e}^{-\frac{(t-{t}_{shift})}{{T}_{1,Pyr}}}\cdot {(cos\theta )}^{\frac{(t-{t}_{shift})}{TR}}\cdot (1-{e}^{-(t-{t}_{shift})\cdot {\rm{\Delta }}{\rho }_{Bic}})$$

At all of the time points, the signal-to-noise ratio of the [1-^13^C]lactate signal was superior to that of [^13^C]bicarbonate signal. For this reason, the t_shift_ value was determined from the [1-^13^C]lactate signal and then applied to the [^13^C]bicarbonate signal.

#### First-order reaction kinetics

The data were also fitted to a first-order reaction kinetics model using an approached developed previously and described in detail by Allouche-Arnon *et al*.^36^.

### Curve fitting

Curve fitting was performed using Matlab (Mathworks, Natick, MA, USA).

### Statistical analysis

Statistical analysis was performed with the tools available in Excel (Microsoft, Redmond, WA, USA).

### Study limitations

#### Brain slices

Brain slices have been in use for about 50 years in the study of brain metabolism and physiology. This preparation is being continuously improved for maintaining viability of the cells in the tissue slices, although in essence this preparation involves an insult to the tissue caused by excision and slicing. It can be seen in Fig. 1 that the brain slices used in the current study do contain viable cells, as evident by ATP and PCr signals. Also, the stability of the PCr/ATP ratio suggests a constant metabolic state at the time of the hyperpolarized experiments. We note that to date, brain slices continue to serve for the study of important and prevalent neurological conditions such as stroke and traumatic brain injury^46^, despite the inherent limitation imposed by the excision and slicing insult, as these systems allows control of the brain microenvironment in a way that is unattainable *in vivo*^47^. For this reason, brain slices are considered as a valuable tool for brain research and have been used in the current study as well.

#### ^31^P NMR spectroscopy as a tool to monitor tissue viability and intracellular pH

First and foremost, ^31^P is useful to demonstrate the brain slices viability by the presence of PCr and ATP. Indeed, the sensitivity in our study is not high due to the use of a relatively low magnetic field (5.8 T), a 10 mm NMR tube, and a 10 mm broadband probe that is not of the highest sensitivity available today. This reduced sensitivity was discussed previously and compared to the sensitivity of a 5 mm NMR tube in a 5 mm probe in an 11.8 T spectrometer^48^. Nevertheless, we use here the same system that is available for hyperpolarized ^13^C experiments with its pros (proximity to the dDNP polarizer, good sensitivity for ^13^C, 10 mm probe – required for 10 mm NMR tube which are needed for the brain slices and the perfusion system) and its con (low sensitivity for ^31^P). Although the ^31^P sensitivity is low, it is sufficient in order to observe the signals of ATP, PCr, and the inorganic phosphate. Indeed, the larger extracellular inorganic phosphate may hide the intracellular inorganic phosphate signal when the pH difference between the two is small. However, larger pH changes, usually acidification of the intracellular space leading to a shift of the intracellular inorganic phosphate signal to a lower field, can be discriminated. By processing ^31^P spectra with a line fitting approach^44^ it can be seen that on the 1^st^ spectrum in Fig. 1, acquired during a recovery period, prior to the hyperpolarized injections, such a shift is visible. This shift is indicative of an intracellular pH of 6.9^33^. It is a testament to the recovery of the slices that the intracellular phosphate signal is not discernible in the following spectra.

Regarding the visibility of the β-ATP signal which is the one signal of the three ^31^P signals of ATP which is solely due to ATP (and not ADP), we note that it is a wider signal (line width at half-height of 68 Hz) compared to α-ATP (34 Hz), γ-ATP (41 Hz), and to PCr (11 Hz). This phenomenon is known in the literature and is likely related to the binding to the divalent magnesium ion, which is essential for the biological activities of ATP. This widening of the signal leads to a lower signal intensity of the β-ATP signal, which in some of our spectra is challenging due to the noise level.

We note that the acquisition conditions that are used for ^31^P acquisition will affect the PCr to ATP ratio as the T_1_ of PCr is much longer than that of ATP^49^. Here, on most experimental days and as shown in Fig. 1, the relatively long repetition time (3.8 s) enabled visualization of a high PCr-to-ATP ratio. On each experimental day the same acquisition parameters were used for ^31^P acquisitions throughout the day and the stability of the PCr-to-ATP was verified.

#### The effect of cumulative pyruvate on energy metabolism in the brain slices

In the studies described herein, the concentration of hyperpolarized pyruvate surrounding the brain slices was 14 mM (with the exception of two injections with 28 mM). This concentration was in the 4 ml of the dissolution media which engulfs the slices following the injection. When the perfusion was returned, this volume was diluted in 200 ml of the main medium reservoir reaching a concentration of 0.27 mM after the 1^st^ injection and 0.54 mM after the 2^nd^ injection, as the injected pyruvate was not washed out of the system. Pyruvate is considered neuroprotective against ischemia and neurotoxicity^32,50,51^. Pyruvate is transported into the brain cells by the monocarboxylate transporter (MCT) family of transporters which transports also lactate and other monocarboxylates^52^. On one hand, MCT3 and MCT4 found in choroid plexus and astrocytes, respectively, transport lactate and pyruvate with a K_m_ of 6 mM and 150 mM, respectively. On the other hand, one of the sodium coupled monocarboxylate transporters (SMCT), SLC5A8, is expressed in brain and is a higher affinity transporter with a K_m_ value for lactate of 159 μM (K_m_ for pyruvate not reported todate, to the best of our knowledge)^52^. Due to the wide range of transport affinities, it is unlikely that the minute amounts of pyruvate left in the aCSF will affect the kinetics of the following injections. Also, the rate of transport cannot be predicated and must be tested experimentally. The degree to which the brain metabolizes pyruvate had been under debate^51^ and this quantification is the topic of the current research. With regard to the energy status of the slices, the minute amounts of pyruvate added to the aCSF after each injection are not likely to affect it. The confirmation for this assumption is likely observed as stable PCr/ATP in the ^31^P NMR spectra acquired prior to and after these pyruvate injections. Specifically, during the maintenance of the slices in the perfusion system (prior to, in between, and after the hyperpolarized injections), since the glucose concentration is 10 mM, the pyruvate produced through glycolysis likely exceeds that supplied by the transport of the minute amounts of pyruvate in the aCSF following the hyperpolarized injections.

#### Non-physiological concentration of pyruvate

During the hyperpolarized measurement, the brain cells are exposed to a high pyruvate concentration which is likely non-physiological. In the future we will aim at decreasing this concentration of pyruvate, although this may not represent zero-order kinetics and will require more complex modelling of the reaction rates. We note, however, that to the best of our knowledge, all hyperpolarized metabolic studies use substrate concentrations that are higher than those present in the tissue under normal healthy physiological conditions.

#### Useful time frames for kinetic analysis and their identification

On each experimental day, one rat was sacrificed to obtain brain slices. The brain slices were then maintained in the perfusion system in the NMR spectrometer and experienced 1–2 hyperpolarized pyruvate injections. We report here only on injections made within a time frame where the PCr/ATP values were not altered. Table 1 displays the entire information for all of the injections studied. However, we found that some injections produced slice movement and then settling back into the NMR probe region. For this reason, not all time frames were useful for kinetic analysis because the change in both the substrate and the products concentration was influenced by this movement and space occupancy in the NMR probe. We believe that the method developed here will be useful for any lab attempting the study of hyperpolarized substrate metabolism in brain slices or other precision cut tissue slices as it provides a means to retrospectively evaluate tissue motion and select the time frame which is appropriate for kinetic analysis.

#### Stress during the hyperpolarized injection

In studies of hyperpolarized substrate metabolism in tissue culture it is customary to stop the perfusion circulation prior to the injection of the hyperpolarized material. This is done in order to allow the maximal time for absorption of the short lived hyperpolarized state of the substrate by the cells and observe metabolism. This action also facilitates the kinetic analysis as the concentration of the substrate is constant. However, most studies that have been performed to date using such perfusion systems were carried out on cancerous tissues which are relatively immune to stress conditions. Here, the study was performed on healthy brain tissue. It is possible that the relatively long time in which oxygen and glucose were not replenished (about 4 min) led to temporary metabolic stress in the slices. We did not detect such stress in the ^31^P phosphate profile but this acquisition averaged 15 and 30 min in which the stressful state was likely resolved. However, the low bicarbonate level determined here may indicate such a temporary stress. In the future we intend to inject glucose in the dissolution media and to oxygenate the dissolution medium to saturation. Nevertheless, we note that the ratio of lactate-to-bicarbonate production rates observed here in brain slices was within the range of their intensity ratio observed *in vivo* (1:5–30)^12,13,15,16,19,20^.

#### Slice thickness

In this study we used precision cut brain slices with 500 μm thickness. This was done to obtain mechanical rigidity of the slices which enables distribution of the slices within the NMR tube rather than a sinking lump at the bottom of the tube. However, it is known that the viability of the cells in the slices is limited by the diffusion such that only the 125 μm closest to the surface receive enough oxygen, glucose, and solutes to maintain viability. In addition, in the ischemic parts, leaching of nucleoside bases occurs, which prohibits ATP recovery^53^. Thus, about 250 μm of each slice depth are viable while the middle is likely dead. Neurotoxins released during stress and necrosis are washed from the slice slowly during the recovery period. Nevertheless, the exact portion of viable cells is not known. In the future we intend to improve the technology by testing the feasibility of performing this work with thinner slices.

#### Temperature

In this study we used a maintenance temperature of 32 °C throughout the experiment. This was done to prevent overheating of the slices, which would lead to loss of viability. It is likely that enzymatic rates are lower than at 37 °C, which is about the rat normal body temperature. In the future, we aim to establish better temperature control by improving the spectrometer’s temperature control unit. We do note however that electrophysiology studies on single slices are routinely performed at 32 °C.

#### Time of brain extraction and slices production

In this study, it took a relatively long time to extract the brain and complete the slicing procedure. Although throughout the process the tissue was immersed in ice-cold aCSF this is considered a long time for working with a McIlwain tissue chopper. In future studies we aim to produce the slices much faster, in less than 2 min. Nevertheless, we note that current electrophysiology done on brain slices also has about 30 min of immersion in ice-cold aCSF while producing the slices in a vibrotome. This is because the vibrotome operates slowly to achieve a cut that is clean with minimal pressure on the brain while slicing. This leads to better visibility under the microscope. We acknowledge the possible loss of some degree of viability prior to the arrival of the slices to the spectrometer due to this prolonged production time.

#### Determination of the relative space occupied by medium and brain slices in the probe

Determination of this ratio is important for (1) calculating the amount of pyruvate visible by the probe, given knowledge on the concentration of pyruvate in the dissolution medium; and (2) determining the wet weight of the tissue in which the observed reaction occurred. Both these factors contribute directly to the calculated rate of metabolite production. In the current study this ratio was estimated based on repeated weighing of tissue slices occupying the probe under the same perfusion conditions. But this estimate is not per hyperpolarized experiment. In the future we aim to improve this determination in order to improve the accuracy of the determination of metabolic rates. One possibility would be to use NMR visible compounds that are limited to only one of the compartments, for example to the extracellular space.

### Data availability statement

The datasets generated and analyzed during the current study are available from the corresponding author on reasonable request.
